# Epidermal growth factor receptor-mutant non-small cell lung Cancer and Choroidal metastases: long-term outcome and response to epidermal growth factor receptor tyrosine kinase inhibitors

**DOI:** 10.1186/s12885-020-07630-6

**Published:** 2020-12-03

**Authors:** Clémentine Bouchez, Johan Pluvy, Ghassen Soussi, Marina Nguenang, Solenn Brosseau, Morgan Tourne, Mégane Collin, Nathalie Théou-Anton, Alice Guyard, Jamila Ammar, Antoine Khalil, Gérard Zalcman, Valérie Gounant

**Affiliations:** 1 Thoracic Oncology Department & CIC1425-CLIP2 Early Phase Cancer Clinical Trials Unit, University Hospital Bichat-Claude Bernard, Medical Faculty, Université de Paris, Hôpital Bichat, APHP, 46 Rue Henri Huchard, 75018 Paris, France; 2 Department of Genetics, University Hospital Bichat-Claude Bernard, Medical Faculty, Université de Paris, Paris, France; 3 Radiology Department, University Hospital Bichat-Claude Bernard, Medical Faculty, Université de Paris, Paris, France; 4 Department of Pathology, University Hospital Bichat-Claude Bernard, Medical Faculty, Université de Paris, Paris, France; 5 Department of Ophtalmology, University Hospital Bichat-Claude Bernard, Medical Faculty, Université de Paris, Paris, France

**Keywords:** Lung cancer, NSCLC, EGFR mutation, Targeted therapy, Tyrosine kinase inhibitors, Choroidal metastases

## Abstract

**Background:**

Choroidal metastases are the most common eye metastatic site. The prevalence of choroidal metastases in NSCLC patients has been reported to vary from 0.2 to 7% in historical series. Although previously reported, little is known about choroidal metastasis in Epidermal Growth Factor Receptor (EGFR)-mutant Non-small cell lung cancer (NSCLC). This study sought to describe the prevalence of choroidal metastases among patients with EGFR-mutated NSCLC and their characteristics, and to estimate their impact on prognosis.

**Methods:**

We conducted a single-center retrospective study including all consecutive metastatic EGFR-mutant NSCLC patients, from Sept. 2015 to Oct. 2018. The EGFR-mutant NSCLC patients were identified via the Department of Genetics’ files. Patients who exhibited choroidal metastases were compared to patients without choroidal metastases. Kaplan-Meier analysis and log-rank test were conducted to assess median overall survival (OS) from diagnosis for the two groups. The study was approved by the IRB as CEPRO number #2020–010.

**Results:**

Prevalence of choroidal metastases in EGFR-mutated NSCLCs was 8.4% (7/83). Five were women, and four current or former smokers. Molecular analysis showed three tumors with exon 19 deletion, three with L858R mutation, and one with complex exon 21 mutation. The choroidal metastases were symptomatic in six/seven patients. Visual disturbances decreased in all but one symptomatic cases upon EGFR TKI, and the choroidal response was maintained over time.

Median follow-up was 42.2 mo (95%CI [37.2–47.1]). Median OS in the choroidal metastasis group was 23.4 mo (95%CI [0.1–51.4]) versus 27.9 mo (95%CI [16.9–38.9]) in the non-choroidal metastasis group (*p* = 0.32). In the choroidal metastasis group, 2-year and 5-year OS were 47.6 and 0%, respectively, versus 55.8 and 26.3% in the non-choroidal metastasis subset.

**Conclusions:**

Choroidal metastases in NSCLC EGFR-mutant patients are rare but should be systematically suspected in case of visual disturbance. TKIs are efficient for treating visual symptoms. Whether choroidal metastases confer a worse prognosis remains unclear owing to the third-generation EGFR TKI osimertinib first-line registration.

**Supplementary Information:**

The online version contains supplementary material available at 10.1186/s12885-020-07630-6.

## Background

Choroidal metastases are the most common eye metastatic site, though the underlying mechanism is poorly understood. The choroid is the vascular, pigmented tissue lying between the sclera and the retina. Its principal role is to supply the retina and part of the optic nerve with oxygen and nutrients, thereby forming a vascular avenue to the sequestered tumor and a favorable environment for tumor growth [1].

In a retrospective study published in 1997 on 950 uveal metastases from 420 patients, the choroid was the most common ocular metastatic site (88%), followed by the iris (9%) and the ciliary body (2%) [1]. Choroidal metastases from lung cancer (21%) were mostly unilateral compared to those occurring in breast cancer (47%) [1]. At diagnosis, the primary cancer was unknown in 34% of cases. Choroidal metastases can thus be inaugural [1].

Revealing symptoms include visual acuity loss, blurred vision, diplopia, and pain [2]. Mass effect and retinal signs, such as retinal detachment, retinal edema, and subretinal fluid, may also be observed.

Diagnosis necessitates ophthalmological expertise, owing to the many examinations required, such as auto-fluorescence, ultrasonography, optical coherence tomography, and fluorescein angiography [2].

Managing choroidal metastases has not been standardized and depends on the systemic disease and number and location of choroidal metastases. The spectrum of treatments extends from systemic therapies to local treatments, such as radiotherapy and intravitreal injection of anti-vascular endothelial growth factor [3, 4].

Non-small cell lung cancer (NSCLC) accounts for 80% of all lung cancer cases, with mutation of the epidermal growth factor receptor (EGFR) found in 10–15% in Caucasian patients [5].

Shimomura et al. first described the resolution of inaugural choroidal metastases, secondary to a lung cancer harboring an exon 19 deletion of the *EGFR* gene, in a woman treated with gefitinib [6].

To the best of our knowledge, the largest described series of choroidal metastases in lung cancer with an *EGFR* gene mutation comprised only two patients [7].

This study sought to describe the prevalence of choroidal metastases among patients with EGFR-mutated NSCLC and their characteristics, and estimate their impact on prognosis.

## Methods

### Patients

We conducted a single-center retrospective study involving a cohort of consecutive metastatic NSCLC patients with EGFR mutations managed between September 2015 and October 2018 at Bichat University Hospital, Paris, France. The EGFR-mutant NSCLC patients were identified via the Department of Genetics’ files, our institutional medical electronic file and drug prescription software. Patient demographics and clinical courses were retrospectively retrieved from medical records. These include: sex, age at diagnosis, history of tobacco smoking, choroidal metastasis status, EGFR mutation subtypes and co-mutation status.

All brain magnetic resonance imaging (MRIs) scans performed at diagnosis for EGFR-mutant patients were reviewed to identify asymptomatic choroidal metastases.

Death was considered the outcome of interest. The time origin was set to the date of the pathological diagnosis and the status of each patient (alive, deceased, or lost to follow) was determined on the study termination date (set to December 18, 2019).

According to the French regulation for observational retrospective studies, all patients received the printed institutional information sheet stating their pseudonymised personal clinical, biological and evolution data could be analyzed for medical and epidemiological studies, but that they keep the possibility to oppose to the utilization of such data. The computed patient study file was registered at the National Commission for Computing Liberties (CNIL registration number #2,161,770), with the study approved by the Institutional Review Board of the French-Learned Society for Respiratory Medicine (Société de Pneumologie de Langue Française) (CEPRO number #2020–010).

### Molecular analysis

Molecular analyses were routinely performed for all non-squamous metastatic NSCLC cases at diagnosis. For liquid biopsy, blood samples were collected into Cell Free DNA collection tube® (Roche) to achieve 4 mL plasma. The Maxwell® 16 FFPE Plus LEV DNA Kit (Promega) and NucleoSnap® DNA Plasma Kit (Macherey-Nagel) were used for DNA and cell-free DNA (cfDNA) extraction, respectively. The DNA and cfDNA quantity were assessed using the Qubit® 2.0 Fluorometer (Qubit® dsDNA BR Assay Kit) and Qubit® DNA HS Assay Kit (Life Technologies-Thermo Fisher Scientific), respectively. The DNA was amplified using primers from Oncomine™ Solid Tumor DNA and Oncomine Solid Tumor plus V2 (Life Technologies-Thermo Fisher Scientific). Usually, more than 200 ng of cfDNA was obtained (median 0.9 ng/μl). The minimum DNA concentration needed to perform the experiments has previously been defined by the manufacter is 20 ng to assure a 0.1% sensitivity with next generation sequencing. The cfDNA was amplified using primers from Oncomine™ Lung cfDNA Assay (Life Technologies-Thermo Fisher Scientific). Sequencing was performed on S5XL System with the Ion 520 or 530 Chip (Ion Torrent, Thermo Fisher Scientific). The sequences were aligned with the hg19 human reference genome, with data analyzed by variant calling using the Torrent Suite software (Thermo Fisher Scientific) and Ion Reporter software for annotations of variants (Thermo Fisher Scientific). Potential mutations were retained on the DNA if their allelic frequency was ≥3% and coverage >300X, and on the cfDNA if their allelic frequency was ≥0.1% and mol count > 2. Mutations were referred to the COSMIC database (GRCh37, COSMIC v84).

The EGFR mutations were classified according to exon location. When next-generation-sequencing (NGS) identified – at diagnosis, prior to EGFR tyrosine kinase inhibitor (TKI) therapy - one or more somatic mutations in other genes (TP53 mutation, KRAS mutation, BRAF mutation, PTEN mutation, DDR2 mutation, CTNNB1 mutation, or PIK3CA mutation) or multiple EGFR mutations, mostly combining an EGFR TKI-sensitizing mutation and uncommon mutation, these situations were defined as “co-mutation” or “complex EGFR mutation,” respectively. Immunohistochemistry (IHC) was performed on initial diagnostic biopsy using anti-Rb clone 4H1 from Cell Signaling Technology (Danvers, MA) at 1:400 dilution, with normal stromal or inflammatory cells serving as internal positive control, in order to determine whether Rb protein expression was lost, likely indicating *RB* gene mutation or deletion.

### Statistical analysis

Categorical variables were expressed as frequencies and percentages. Student T test was used for independent samples to compare means, after checking the data follow a normal distribution (tested by Shapiro-Wilk method). Mann-Whitney U test was used instead when the data was not normally distributed. Fisher’s exact test was used to compare percentages. The median follow-up was evaluated using the reverse Kaplan-Meier estimator. The impact of each variable on survival was studied in univariable analysis using the Kaplan–Meier method. The log-rank test was used to compare survival distributions between groups. At that step, hypothesis testing was two-tailed, and *p*-values were set at < 0.05 for statistical significance. The hazard ratios (HR) and their respective 95% confidence intervals (95%CI) were calculated from univariable analyses using Cox proportional hazards models. Multivariable Cox analysis was performed using backward stepwise regression. The tested variables in univariate overall survival (OS) analysis were age, sex, smoking status, choroidal metastasis status, EGFR mutation subtype, co-mutation status, complex EGFR mutations, and TP53 co-mutation. Only variables with a significance threshold *p* ≤ 0.20 in univariate analysis were included as candidate variables in the modeling procedure. Choroidal metastasis status was forced into the multivariate model, whatever was its *p*-value in the univariate analysis. Statistical analyses were performed using IBM SPSS Statistics for Windows, version 25.0 (IBM Corp., Armonk, N.Y., USA).

## Results

### Patient demographics and characteristics in the whole study population (Table 1)

We identified 83 cases of metastatic NSCLC harboring an EGFR mutation (Table 1) in our electronic files during a three-year period. Of these, 20 cases were diagnosed earlier but referred to our Department for treatment during this time period. The other 63 were newly diagnosed NSCLC cases. Main clinical baseline characteristics are summarize in Table 1.

**Table 1:** Baseline Characteristics of Patients with Non-Small-Cell Lung Cancer EGFR Mutation by Choroidal Metastasis Status

Variables	All *n* = 83	Choroidal metastases *n* = 7	Non-choroidal metastases *n* = 76	*p-*value
**Age, years**				
Mean	69	56	70	< 10^−6^
Median (IQR)	69 (60–80)	57 (50–60)	71 (61–80)	
**Sex** (%)				
Male	37 (45)	2 (29)	35 (46)	0.45
Female	46 (55)	5 (71)	41 (54)	
**Smoking status** (%)				
Current smoker	4 (5)	0 (0)	4 (5)	0.79
Former smoker	35 (42)	4 (57)	31 (41)	
Never smoker	44 (53)	3 (43)	41 (54)	
**Subtype of EGFR mutations** (%)				
Exon 19	37 (44)	3 (43)	34 (45)	1
Exon 21	33 (40)	3 (43)	30 (39)	
Others	13 (16)	1 (14)	12 (16)	
**Co-mutation status** (%)				
Yes	52 (62)	5 (71)	47 (62)	1
No	29 (35)	2 (29)	27 (35)	
Unknown	2 (2)	0	2 (3)	
**Complex EGFR mutations** (%)				
Yes	11 (13)	2 (29)	9 (12)	0.36
No	70 (84)	5 (71)	65 (85)	
Unknown	2 (2)	0	2 (3)	
**TP53 co-mutation** (%)				
Yes	40 (48)	4 (57)	36 (47)	0.76
No	41 (50)	3 (43)	38 (50)	
Unknown	2 (2)	0	2 (3)	

*IQR* interquartile range

*EGFR* epidermal growth factor receptor

**Fig. 1:**
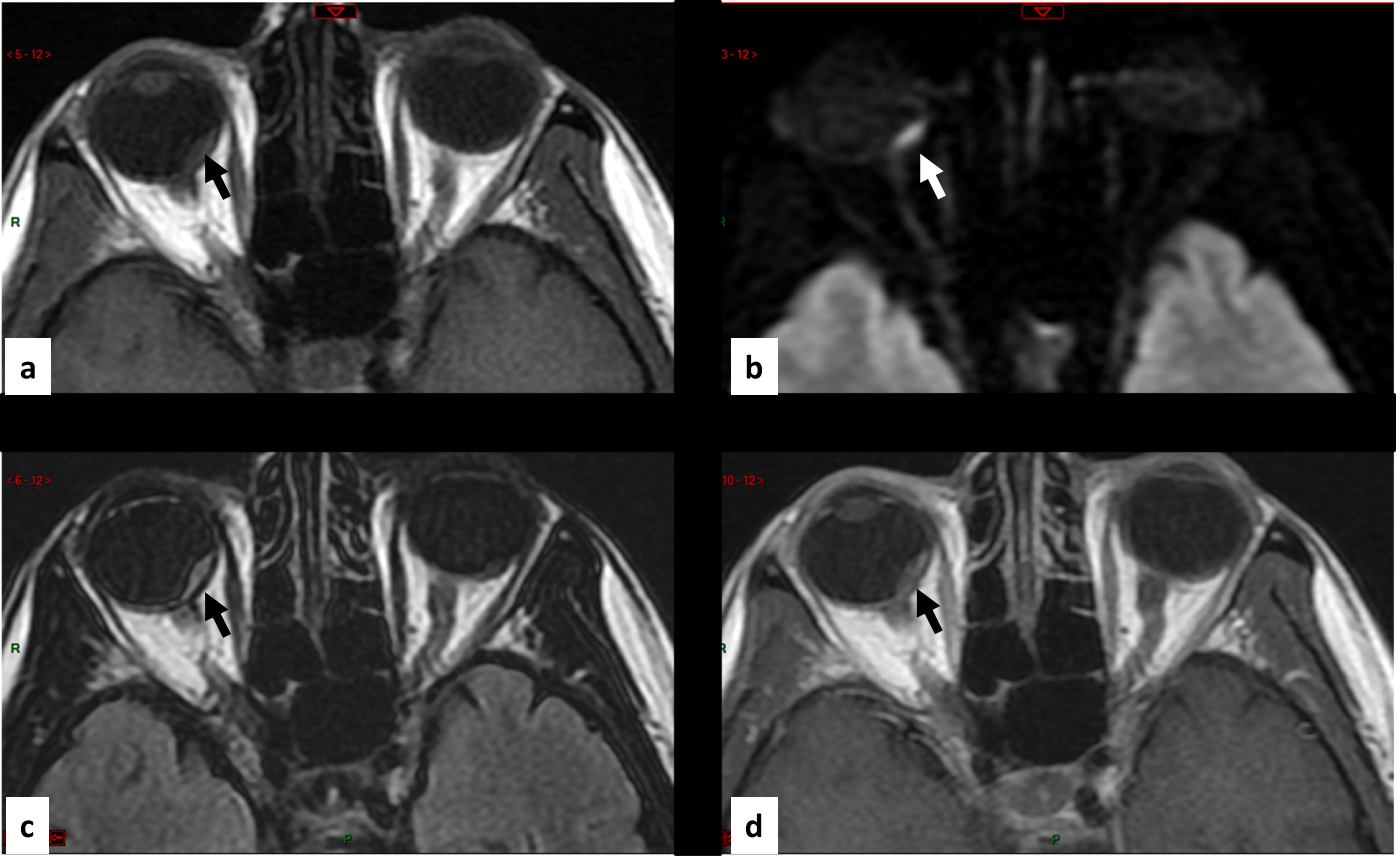
MRI In axial plane focused on orbits with the following sequences in Patient #7. *)* Spin-Echo T1-weighted image, it shows an oblong intraorbital lesion (arrow) in slight hypersignal within the orbit. **b** Diffusion-weighted image, it shows a frank hypersignal of the lesion (arrow). **c** FLAIR, it shows the lesion in slight hypersignal (arrow). **d** Spin-Echo T1-weighted image after contrast injection, it shows a slight enhancement of the intraorbital lesion (arrow)

Among the 83 EGFR-mutant NSCLC patients, six exhibited choroidal metastases, revealed by visual disturbances. An additional case (Fig. 1) was identified upon a retrospective systematical analysis of all brain MRIs performed by an expert radiologist, resulting in a final number of 7 choroidal metastasis patients (8.4%). This latter patient had no clinical visual disturbances.

### Tumor genetic profile in the whole study population (Table 1)

Thirty-seven (44%) tumors harbored exon 19 mutations (either 32 (38%) deletions, and 5 (6%) deletion–insertions). Overall, 33 (40%) tumors had a mutation in *EGFR* exon 21, (31 [38%] L858R, and two [2%] L861Q). Four (5%) tumors exhibited a missense mutation in exon 18, and nine (11%) a mutation in exon 20.

We found 11 tumors (13%) with EGFR complex mutations associated with common or uncommon mutations, and two had two rare mutations associated together. Two tumors (2%) presented with a T790M mutation in exon 20 of the *EGFR* gene at diagnosis, associated with a common activating mutation. Co-mutations involving the *TP53* and *EGFR* genes were found in 40 tumors (48%).

Furthermore, 4/83 (5%) patients experienced SCLC transformation during disease evolution, involving 3/76 (4%) patients without choroidal metastasis, and 1/7 (14%) with choroidal metastasis (Patient #5). These four tumors harbored TP53 mutation at diagnosis.

### Clinical and molecular characteristics of Choroidal metastasis patients (Table 2 and Table 3)

**Fig. 2:**
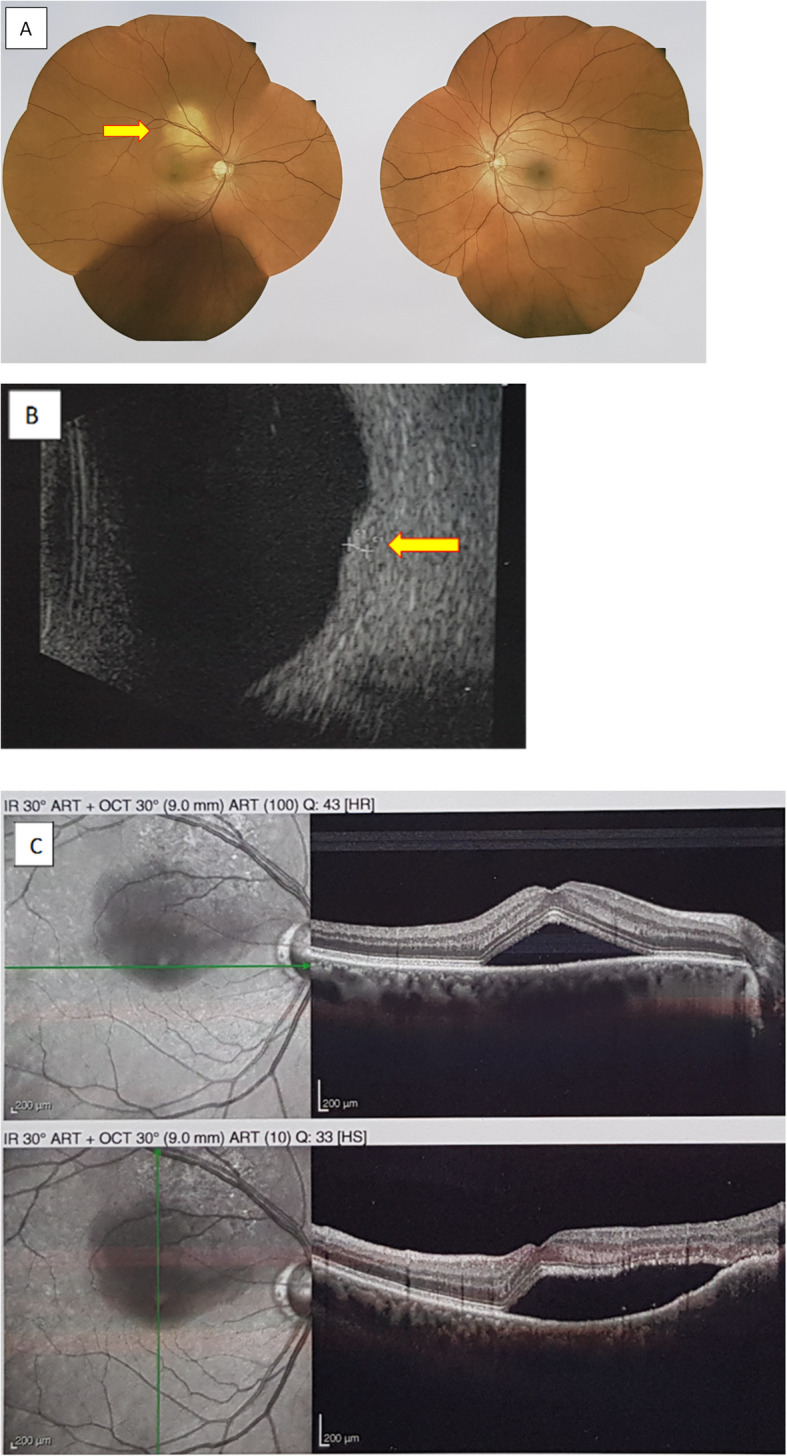
Initial presentation of right choroidal metastasis in Patient #1. **a** Fundus examination showing well-defined yellowish-colored circular submacular lesion on the right eye (arrow). **b** B-scan echography showing a right dome-shaped lesion on macula with hyperreflectivity and a maximal elevation of 1,12 mm (arrow). **c** Fluorescein angiography showing retinal detachment with the presence of subretinal fluid on the right eye

Seven patients (8.4%) with choroidal metastases associated with EGFR-mutant NSCLC were retrieved in our retrospective cohort. Their clinical characteristics at diagnosis are shown in Table 2, while the choroidal metastasis features and their evolution are summarized in Table 3. Initial presentation of choroidal metastasis in Patient #1 is shown in Fig. 2.

**Table 2:** Clinical and Molecular Characteristics of Seven Patients With Choroidal Metastases

Patients	Patient #1	Patient #2	Patient #3	Patient #4	Patient #5	Patient #6	Patient #7
**Sex**	Male	Female	Female	Female	Female	Female	Male
**Ethnic group**	Moroccan	Caucasian	Caucasian	Caucasian	Chinese	Guinean	Moroccan
**Age-ranges at the time of diagnosis**	60–65 years	60–65 years	45–50 years	50–55 years	45–50 years	50–55 years	50–55 years
**History of smoking**	12 pack-years, quitted 18 years ago	27 pack-years, quitted 27 years ago	No	20 pack-years (active)	No	No	30 pack-years (active)
**TNM stage at time of diagnosis (8th classification)**	cT3N0M1c	cT1cN3M1c	cT4N3M1c	cT4N3M1c	cT2N3M1c	cT3N2M1c	cT4N2M1c
**EGFR mutation(s) at time of diagnosis**	Deletion of exon 19 c2236_2250del	Deletion of exon 19 c2240_2257del	Complex mutation of exon 21 Leu833Val His835Leu	Mutation of exon 21 L858R Mutation of exon 20 T790M (AF = 11.6%)	Mutation of exon 21 L858R	Mutation of exon 21 Leu858R	Deletion of exon 19 c2235_2249del
**Mutation TP53**	No	Exon 5	No	Exon 5	Exon 7	No	Exon 7
**IHC Rb**	Positive	Negative	Positive	Positive	Negative	Negative	Negative
**Anti-EGFR treatments (number of lines)**	1	3	2	3	1	1	3
**Anti-EGFR** **TKIs**	Afatinib L1	Afatinib L1 Osimertinib L3 Osimertinib L5	Afatinib L6 Osimertinib L7	Erlotinib L1 Osimertinib L2 Osimertinib L5	Erlotinib L1	Erlotinib L2	Erlotinib L1 Osimertinib L2 Afatinib L4
**Carcinomatous meningitis during disease course**	No	Confirmed by cytology	High MRI probability	High MRI probability	No	Confirmed by cytology	High MRI probability
**Transformation in SCLC during disease course**	No	No	No	No	Yes	No	No
**Alive on Aug. 2020**	Yes	No	No	No	No	No	No
**Follow-up from diagnosis**	25 months	Death at 17 months	Death at 23 months	Death at 27 months	Death at 6 months	Death at 2 months	Death at 36 months

*EGFR* epidermal growth factor receptor, *IHC* immunohistochemistry, *TKI* tyrosine kinase inhibitor, *SCLC* small cell lung cancer, *MRI* magnetic resonance imaging, *AF* allelic frequency

**Table 3:** Diagnosis Characteristics of Choroidal Metastases in Seven Patients

Patients	Patient #1	Patient #2	Patient #3	Patient #4	Patient #5	Patient #6	Patient #7
**Symptoms**	Yes	Yes	Yes	Yes	Yes	Yes	No
**Time to onset of symptoms (months)**	0	0	0	0	0	0	
**Nature of symptoms**	Myodesopsia and visual acuity loss of right eye	Intermittent visual blur on right eye	Visual acuity loss of left eye	Visual acuity loss of right eye	Blurred vision in right eye	Retro-ocular pain and visual acuity loss on right eye	
**Associated metastatic site (yes / no)**	Yes	Yes	Yes	Yes	Yes	Yes	Yes
**Associated brain metastases**	No	Yes	No	Yes	Yes	Yes	Yes
**Diagnostic tool**	Fundoscopic examination Fluorescein angiography B-scan ultrasound	Fundoscopic examination Fluorescein angiography B-scan ultrasound	B-scan ultrasound	Fundoscopic examination Fluorescein angiography B-scan ultrasound	Fundoscopic examination Fluorescein angiography B-scan ultrasound	B-scan ultrasound	Retrospective MRI analysis
**Uni- or bilateral lesion**	Unilateral	Unilateral	Unilateral	Unilateral	Bilateral	Unilateral	Unilateral
**Visibility on MRI (yes / no)**	Not performed	No	Yes	Yes	Yes	Yes	Yes
**Response to systemic therapy**	Objective response to afatinib	Objective response to afatinib	Objective response to chemotherapy	Objective response to osimertinib	No	Not evaluable	Objective response to erlotinib
**Radiotherapy of choroidal metastases**	No	No	No	No	Yes	No	No
**Concomitant progression to visceral progression**	No	No	No	No	No	Not evaluable	No

*MRI* magnetic resonance imaging

### Patient demographics and characteristics of Choroidal metastasis patients

When comparing the clinical characteristics of the choroidal metastasis group with the remaining 76 patients, no statistically significant differences were found, except for a lower mean age in the choroidal metastasis group, (*p* <  10^− 6^). Most of the choroidal metastasis patients were women (five/seven cases, 71%), and the majority (four of seven cases, 57%) were current or former smokers, with two being active smokers. There was only one Asian patient, with three being of African origin.

In all patients (except Patient #7, with retrospective MRI-based diagnosis), the choroidal metastases resulted in symptoms, such as pain, blurred vision, or visual acuity loss. The choroidal metastases were frequently unilateral (six of seven cases, 85%). Brain MRI was performed at diagnosis in six out of seven patients, with choroidal metastases actually visible on MRI after a retrospective secondary analysis by an expert radiologist in five out of six patients, though all these lesions were missed on the first MRI report, except for those of Patient #6.

The choroid metastases were associated with other metastatic sites like brain metastases in five out of seven patients without any obvious leptomeningeal carcinomatosis on brain MRI. Four out off five evaluable patients who were treated with TKI showed an objective response with visual symptom improvement. Local response was maintained in all cases, and no choroidal local progression occurred, even in case of tumor progression at other metastatic sites. Four out of seven patients received osimertinib as second-line therapy based on the emergence of T790M resistance mutation. All patients received a pemetrexed/platinum-based chemotherapy doublet during the course of the disease. All lines of treatments are available in Supplementary Data 1. One patient out of seven also underwent radiotherapy of the choroidal metastasis.

### Genetic profile of EGFR-mutated NSCLC of Choroidal metastasis patients

The molecular tumor status of the choroidal metastasis patients was consistent with that of the remaining population, as 85% had a common activating EGFR mutation (Table 1). One patient had a rare, complex exon 21 mutation diagnosed late in the NSCLC course, using whole exome sequencing.

One patient displayed a T790M mutation in the *EGFR* gene exon 20 at diagnosis at the same allele frequency as the common exon 21 mutation (Patient #4, L858R mutation). The tumor molecular patterns of the seven patients with choroidal metastases are shown in Table 2: four patients had TP53 mutations, four had lost Rb immunohistochemical expression, indicative of a Rb probable gene deletion, and three had simultaneously both events at diagnosis supporting the preexistence of a sub-clone with small-cell carcinoma features. However, during the study period of time, only one case of SCLC histological transformation was observed in one out of seven patients (Patient #5), who similarly exhibited TP53 mutation and Rb loss expression in the diagnostic sample.

### Overall survival analysis (Fig. 3)

Median follow-up time in the whole study population was 42.2 mo., 95%CI [37.2–47.1]. On the study termination date, 28 patients (33.7%) were still alive and 55 patients (66.3%) were deceased. No patients were lost to follow-up.

**Fig. 3:**
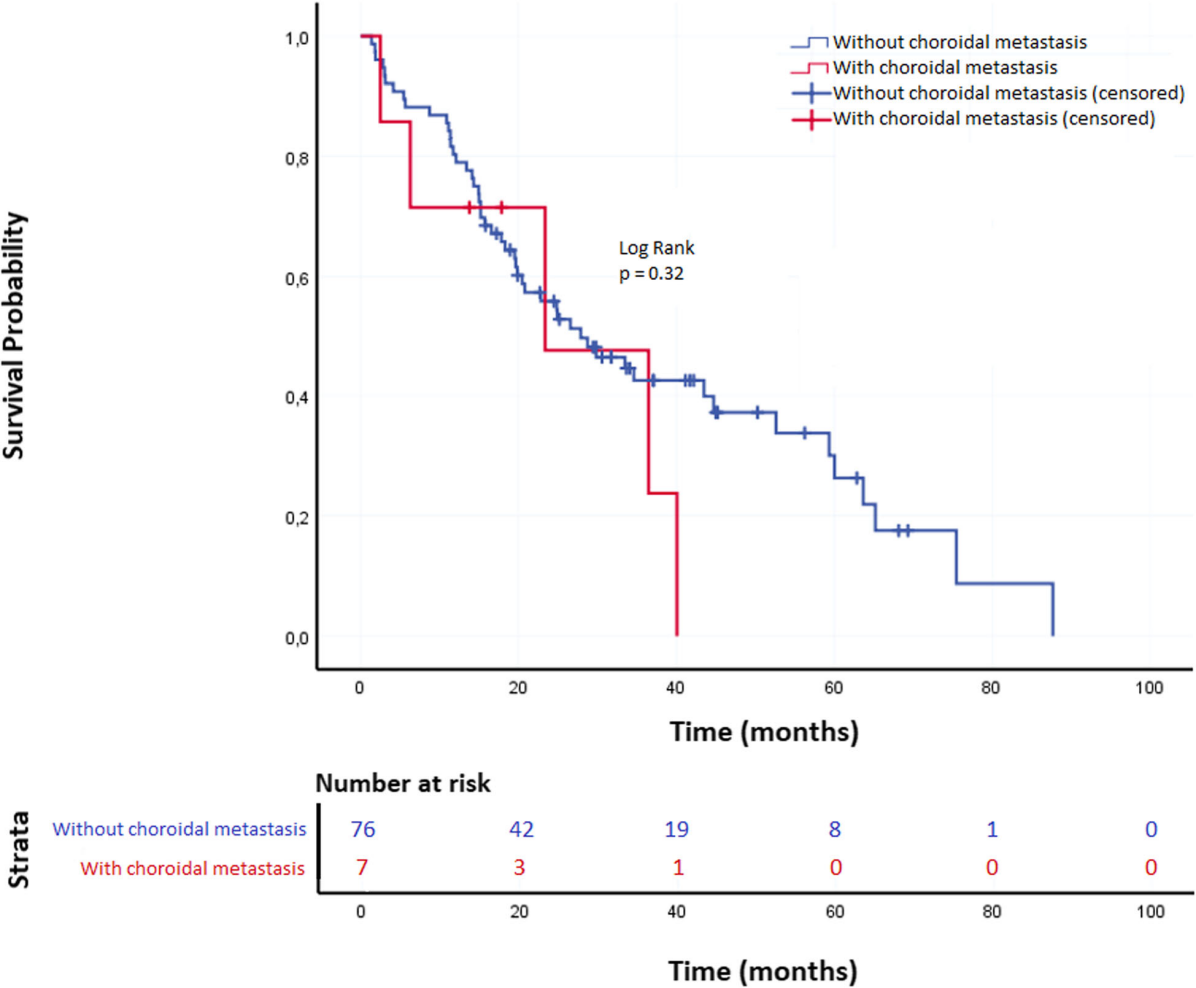
Kaplan-Meier plots showing overall survival of patients according to choroidal metastases status

Median OS in the whole study population was 27.9 mo. (95% CI [17.1–38.6]) with 55.3 and 24.1%, 2-year and 5-year OS, respectively.

In univariable analysis, median OS in the choroidal metastasis group was 23.4 mo. 95%CI [0.1–51.4]) versus 27.9 mo. 95%CI [16.9–38.9] in the non-choroidal metastasis group (*p* = 0.32) (Fig. 3). In the choroidal metastasis group, 2-year and 5-year OS were 47.6 and 0%, respectively. In the non-choroidal metastasis group, 2-year and 5-year OS were found to be 55.8 and 26.3%, respectively. The presence of EGFR exon 19 deletion was the only variable significantly associated with a longer median OS (52.6 mo., 95%CI [24.1–81.1]) as compared with patients showing EGFR exon 21 point mutation (26.6 mo., 95%CI [15.3–37.9]) or non-conventional EGFR mutations (19.8 mo., 95%CI [15.3–24.4]), *p* = 0.03.

After adjusting for variables showing a *p*-value ≤0.02 in the univariate analysis, i.e. EGFR mutation subtype, and TP53 mutations, choroidal metastasis did not show any impact on survival (Supplementary data 2) while EGFR mutation subtype remained as an independent prognostic factor, since patients with exon 21 and uncommon EGFR mutations had significantly shorter survivals, with adj. HR =1.89, 95%CI [1.01–3.55] (*p* < 0.05), and 2.42 95%CI [1.11–5.31] (p = 0.03), respectively, as compared with patients whose tumor harbored EGFR exon 19 deletion.

## Discussion

The specific prevalence of choroidal metastases in EGFR-mutant NSCLC patients is still unknown. Herein, we report the characteristics of seven patients with choroidal metastasis from a cohort of 83 consecutive EGFR-mutant NSCLC patients (8.4%). Based on published data, the prevalence of choroidal metastases in NSCLC patients has been reported to vary from 0.2% [1, 8] to 7% [9] in historical series, reaching 10% in post-mortem histopathological studies [10]. We take charge of nearly 300 new metastatic lung cancer patients each year in our department, pertaining to a 945-bed tertiary University Hospital in the North of Paris, covering a population of roughly 4.0 million inhabitants. Thus, 900 Stage IV lung cancer patients were treated during the same time period. Using the key terms “choroidal metastasis” in our database, we have identified 10 cases of lung cancer with choroidal metastasis over this period: 7 adenocarcinoma harboring EGFR mutations (subject of this study), 1 case with BRAF V600E mutation and 2 cases K-Ras mutated (including one case associated with STK11 mutation).

Among the seven patients with choroidal metastases in EGFR-mutant NSCLC, six were symptomatic, which was concordant with Shields’ cohort of 420 patients with various primary cancers over a 20-year period [1], of whom most were symptomatic. The choroidal metastases were inaugural in these six symptomatic cases, while being a revealing symptom in the first patient described.

Our seven-patient series only differed in age from the non-choroidal metastases population, showing a younger mean age. Most choroidal metastases concerned women, which is not unexpected taking account for the larger female prevalence in patients with EFGR-mutant tumor. However, if lung cancer is the most common source of choroidal metastases in males (40%), it is the second most common etiology in females, after breast cancer (68%) [1].

Concomitant TP53 mutation and Rb expression loss were unlikely to be predictors of choroidal metastasis, although it has been previously associated to SCLC histological transformation, since only three out of seven patients showed TP53/Rb double alterations with only a single patient exhibiting SCLC transformation upon EGFR TKI in our series.

In the United States, the frequency of EGFR testing was reported to be only 22% for Stage IV lung adenocarcinoma in the whole country in 2010 [5], though the guidelines recommend systematic testing for all Stage IV lung adenocarcinomas [11]. Although the rate of EGFR testing may have improved meanwhile, the occurrence of choroidal metastases should strongly support EGFR testing, considering EGFR TKI efficacy. 

Our patients had at least one more metastatic site, which is consistent with Kreusel et al.’s series, in which the only risk factor of developing choroidal metastases in univariate analysis was the presence of at least two other metastatic sites [9].

In our series, a systematic control ophthalmological examination upon EGFR-TKI treatment was not performed and we could not correlate symptom decrease and choroidal lesion disappearance on dilated fundus ophthalmoscopy. Conversely, sub-retinal detachment, on echography or fluorescein angiography, frequently disappeared.

A retrospective analysis of brain MRI by an expert radiologist, who was blinded to the choroidal metastasis diagnosis, identified choroidal metastases in five patients, with a previously known diagnosis in four, and a previously unknown in the remaining one. The choroidal metastases’ appearance upon MRI consisted of a well-demarcated mass iso-intense on T1-weighted images and hypointense on T2-weighted images [12]. Indeed, MRI has proven useful in differentiating choroidal metastases from primary uveal neoplasm, especially when ophthalmological visualization is obscured by subretinal effusion [13, 14]. Since brain imaging is essential to the diagnostic assessment of Stage IV NSCLC, a specific examination focused on the eyes should be recommended, particularly when EGFR-mutant lung cancer patients experience visual symptoms, such as visual disturbance.

Among four out of five evaluable patients treated by EFGR TKI, this treatment yielded a dramatic response with diminution and even disappearance of visual troubles after a 1-month course. Conversely, one patient experienced regression of visual troubles upon chemotherapy prior to TKI initiation. Tumor progression secondary to TKI resistance was not associated with choroidal progression, and choroidal response to TKIs appeared to be maintained, even in case of extra-choroidal progression.

The first described choroidal metastasis cases in possibly EGFR-mutant Asian patients (not molecularly assessed), published in 2009–2010, revealed the efficacy of EGFR TKI (in association with intravitreal bevacizumab) [15, 16]. Since then, a few case reports have confirmed the ability of first- or second-generation TKIs to induce choroidal metastasis regression in patients with tumor EGFR mutation [7, 17, 18]. The efficacy of third-generation EGFR TKI osimertinib on choroidal metastasis was reported once in an NSCLC patient with T790M EGFR tumor mutation [19]. Our series consisted of patients treated before first-line setting registration of osimertinib, though four out of seven received osimertinib as post-front-line treatment. Owing to osimertinib efficacy on brain metastases demonstrated in large Phase 3 trials in patients with or without T790M mutation [20, 21], osimertinib should also be considered the first-line treatment of choice in EGFR-mutant lung cancer patients with choroidal metastases.

The specific prognosis of EGFR-mutated lung cancer with choroidal metastases is unknown. In a study by Shah [22] involving 194 lung cancers (NSCLC and SCLC) with uveal metastatic tumors, the 1-year mortality from eye diagnosis time was estimated at 54%, with a median survival < 12 months. In our series, the median OS in patients with choroidal metastases was 23.4 months (95%CI [0.1–51.4]) versus 27.9 months (95%CI [16.9–38.9]) in those without choroidal metastases, in line with the results of both first-line osimertinib Phase 3 trial [21], in Stage IV patients with classical EGFR-activating mutations, and the second-line osimertinib AURA3 Phase 3 in patients with T790M resistance mutations [20]. Therefore, the specific prognostic role of choroidal metastases remains questionable.

In addition to its single-center nature, a major limitation of our study is its small sample size, accounting for its low statistical power and unmeasured confounders. However, as exon 19 mutations were an independent factor of OS in our cohort, it is probably representative of a Caucasian EGFR-mutant population.

It must be mentioned that choroidal metastasis is a rare clinical condition, encountered in 1–8% of the whole EGFR-mutant NSCLC population. We feel that our observations could support larger-scale prospective surveys on a national or international scale.

## Conclusions

In conclusion, choroidal metastases in NSCLC EGFR-mutant patients are rare but should be systematically suspected in case of visual disturbance. TKIs are efficient for treating visual symptoms in such EGFR-mutant patients. Once obtained, choroidal response seems to be maintained, even when visceral tumor progression is observed. Whether choroidal metastases confer a worse prognosis remains unclear owing to the third-generation EGFR TKI osimertinib first-line registration.

## Supplementary Information

**Additional file 1:** **Supplementary data 1.** All lines of treatment of Seven Patients With Choroidal Metastases

**Additional file 2:** **Supplementary data 2.** Univariable and Multivariable Analysis for Overall Survival in EGFR-Mutated Advanced NSCLC patients

## Data Availability

The datasets used and/or analysed during the current study are available from the corresponding author on reasonable request.

## Abbreviations

AF: Allelic Frequency
cfDNA: cell-free DNA
CI: Confidence Interval
EGFR: Epidermal Growth Factor Receptor
FDG: Fluorodeoxyglucose
FISH: Fluorescence In Situ Hybridization
HR: Hazard Ratio
IHC: Immunohistochemistry
MRI: Magnetic Resonance Imaging
NGS: Next-Generation-Sequencing
NSCLC: Non-Small Cell Lung Cancer
OS: Overall Survival
PET-CT: Positron emission Tomography-CT
SCLC: Small Cell Lung Cancer
TKI: Tyrosine Kinase Inhibitors
TPS: Tumor Proportion Score
