# Supplementary material for: Epidermal growth factor receptor-mutant non-small cell lung Cancer and Choroidal metastases: long-term outcome and response to epidermal growth factor receptor tyrosine kinase inhibitors
Source: BMC Cancer. 2020 Dec 3;20:1186. doi: 10.1186/s12885-020-07630-6 (PMC7712981; doi:10.1186/s12885-020-07630-6)
Supplement: Supplementary file 1 — Additional file 1: Supplementary data 1. All lines of treatment of Seven Patients With Choroidal Metastases [file 12885_2020_7630_MOESM1_ESM.docx]

**Supplementary data 1.** All lines of treatment of Seven Patients With Choroidal Metastases

| **Patients** | **Patient #1** | **Patient #2** | **Patient #3** | | **Patient #4** | **Patient #5** | **Patient #6** | **Patient #7** |
| --- | --- | --- | --- | --- | --- | --- | --- | --- |
| **Treatment (number of lines)** | 2 | 5 | | 8 | 5 | 4 | 2 | 4 |
| **Anti-EGFR treatments (number of lines)** | 1 | 3 | | 2 | 3 | 1 | 1 | 3 |
| **Treatment** | Afatinib L1  Carboplatine- Pemetrexed L2 | Afatinib L1  Osimertinib L2  Crizotinib L3  Cisplatine- Pemetrexed- Bevacizumab L4 Osimertinib L5  Paclitaxel L6 Carboplatine- Pemetrexed L7 | | Carboplatine-Pemetrexed L1  Nivolumab L2  Paclitaxel-Bevacizumab L3  Gemcitabine L4  Docetaxel L5  Afatinib L6  Osimertinib L7  Carboplatine-Pemetrexed L8 | Erlotinib L1  Osimertinib L2  Carboplatine-Pemetrexed L3  Carboplatine-Paclitaxel-Bevacizumab L4  Osimertinib L5 | Erlotinib L1  Cisplatine- Pemetrexed L2  Carboplatine- Paclitaxel- Erlotinib L3 | Carboplatine- Pemetrexed L1  Erlotinib L2 | Erlotinib L1  Osimertinib L2  Carboplatine- Pemetrexed L3  Afatinib L4  Alectinib L5 |

EGFR, epidermal growth factor receptor; TKI, tyrosine kinase inhibitor.
