# Supplementary material for: Epidermal growth factor receptor-mutant non-small cell lung Cancer and Choroidal metastases: long-term outcome and response to epidermal growth factor receptor tyrosine kinase inhibitors
Source: BMC Cancer. 2020 Dec 3;20:1186. doi: 10.1186/s12885-020-07630-6 (PMC7712981; doi:10.1186/s12885-020-07630-6)
Supplement: Supplementary file 2 — Additional file 2: Supplementary data 2. Univariable and Multivariable Analysis for Overall Survival in EGFR-Mutated Advanced NSCLC patients [file 12885_2020_7630_MOESM2_ESM.docx]

**Supplementary data 2.** Univariable and Multivariable Analysis for Overall Survival in EGFR-Mutated Advanced NSCLC patients

|  | **Univariable analysis** |  | **Multivariable analysis** |  |
| --- | --- | --- | --- | --- |
|  | HR (95% CI) | p*-*value | aHR (95% CI) | p-value |
| **Age, years**  < 65  ≥ 65 | 1 [reference]  1.30 (0.74-2.26) | 0.36 | **-----** | **-----** |
| **Sex**  Female  Male | 1 [reference]  1.34 (0.78-2.30) | 0.29 | **-----** | **-----** |
| **Smoking status**  Former or current smoker  Never smoker | 1 [reference]  1.34 (0.78-2.30) | 0.29 | **-----** | **-----** |
| **Choroidal metastasis**  No  Yes | 1 [reference]  1.60 (0.63-4.06) | 0.32 | **-----** | **-----** |
| **Subtype of EGFR mutation**  Exon 19  Exon 21  Others | All subtypes  1 [reference]  2.08 (1.11-3.86)  2.32 (1.07-5.06) | **0.04**  **0.02**  **0.03** | 1 [reference]  1.89 (1.01-3.55)  2.42 (1.11-5.31) | **<0.05**  **<0.05**  **0.03** |
| **Co-mutation status**  Yes  No | 1 [reference]  1.32 (0.76-2.30) | 0.33 | **-----** | **-----** |
| **TP53 co-mutation**  Yes  No | 1 [reference]  1.53 (0.88-2.65) | 0.13 | 1.60 (0.92-2.80) | 0.10 |

HR, hazard ratio; CI, confidence interval; EGFR, epidermal growth factor receptor.
